# Influence of HA on Release Process of Anionic and Cationic API Incorporated into Hydrophilic Gel

**DOI:** 10.3390/ijms24065606

**Published:** 2023-03-15

**Authors:** Dorota Wójcik-Pastuszka, Karolina Stawicka, Andrzej Dryś, Witold Musiał

**Affiliations:** Department of Physical Chemistry and Biophysics, Faculty of Pharmacy, Wrocław Medical University, ul. Borowska 211A, 55-556 Wrocław, Poland

**Keywords:** hydrogels, sodium hyaluronate, lidocaine hydrochloride, sodium naproxen, release, kinetics, FTIR study, DSC study

## Abstract

The properties of sodium hyaluronate (HA), such as hygroscopicity, flexibility, the ability to form hydrogels, as well as biocompatibility and biodegradability, are beneficial for the applications in pharmaceutical technology, cosmetics industry, and aesthetic medicine. The aim of this study was to prepare HA-based hydrogels doped with active pharmaceutical ingredient (API): a cationic drug—lidocaine hydrochloride or anionic drug—sodium. The interaction between the carrier and the implemented active pharmaceutical substances was evaluated in prepared systems by applying viscometric measurements, performing release tests of the drug from the obtained formulations, and carrying out FTIR and DSC. The data from release studies were analyzed using the zero-, first-, and second-order kinetics and Higuchi, Korsmeyer-Peppas, and Hixon-Crowell models. The respective kinetic parameters: the release rate constants, the half-release time and, in the case of the Korsmeyer-Peppas equation, the n parameter were calculated. The variability between the obtained release profiles was studied by calculating the difference (f_1_) and the similarity factor (f_2_) as well as employing statistical methods. It was revealed that the incorporation of the drugs resulted in an increase in the viscosity of the hydrogels in comparison to the respective drug-free preparations. The dissolution study showed that not entire amount of the added drug was released from the formulation, suggesting an interaction between the carrier and the drug. The FTIR and DSC studies confirmed the bond formation between HA and both medicinal substances.

## 1. Introduction

Hydrophilic gels are a group of semi-solid preparations applied to the skin, mucous membranes, and wounds. The hydrogel base consists of water and synthetic or natural polymer. Sodium hyaluronate (HA) belongs to a class of biopolymers that can be used for the preparation of hydrogels. An average human body of 70 kg contains about 15 g of HA, mostly in the skin or in the intercellular space [1]. The HA of high molecular weight has beneficial properties in terms of skin health, including improvement of the nutrition, support of regeneration of the cells, reparation, and the rebuilding of the epidermis [2]. The regenerating, nourishing, and moisturizing properties of HA, as well as its natural occurrence in dermal tissues, results in wide use in the cosmetics industry, aesthetic medicine, and the pharmaceutical industry as a drug carrier. The medical and pharmaceutical applications are effective due to the biocompatibility, good rheological properties, and biodegradability of HA [3,4].

**Figure 1:**
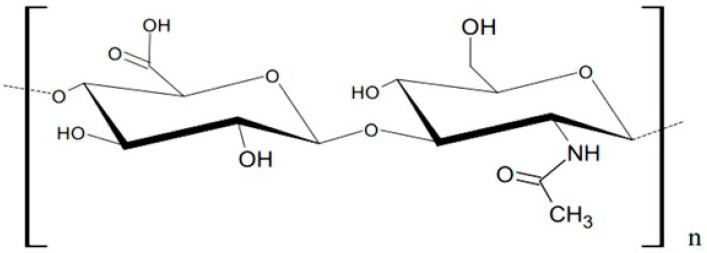
The structure of hyaluronic acid disaccharide.

HA is a polymer composed of disaccharide units consisting of alternating molecules of D-glucuronic acid and N-acetyl-D-glucosamine (Figure 1) [5].

The molecular weight of a single disaccharide is about 400 Da. In one HA chain, there are up to 25,000 disaccharide fragments, resulting in HA molecular weight of ca. 10 MDa. The carboxyl group of hyaluronic acid may undergo dissociation, sodium cation attachment and the formation of sodium hyaluronate. The hydroxyl groups in the disaccharide molecule provide good water solubility and bind large amounts of water molecules to form a viscous hydrogel. The hygroscopic properties of HA ensure good skin hydration [6,7]. The interactions of HA with various molecules provide the scaffold for the pericellular matrix and ensure an environment for cell proliferation and migration, which are especially important during wound healing [8]. The high-molecular HA present in the body has an anti-inflammatory effect; however, the short-chains of HA intensify the inflammation [7].

Gupta et al. [9] revealed that the concentration and the molecular weight of HA are not stable in the body. HA decays due to diseases and age. Moreover, it may decompose under the influence of other factors such as ultrasound, pH, temperature, and enzymes. During ultrasound treatment, a relationship between the molecular weight of HA and time was observed. It has been noticed that the high molecular weight HA breaks down more slowly than the low molecular weight HA. The molecular weight of HA present in the body is in the range of 10^4^–10^5^ Da and depends on the internal source [10,11].

The viscosity of aqueous dispersions of HA increases with increasing HA concentrations. The resulting gel, unmodified and non-cross-linked, is usually quickly removed from the body by the hyaluronidase enzymes [6,12]. In order to increase the stability of the HA-based hydrogel, cross-linkers are added to form a durable gel resistant to enzymes and free radicals. The disintegration of such gel is much slower, and the preparation remains longer in the place of application, e.g., in the skin. The hardness and topical residence time of the preparation increase with the cross-linking degree. However, there is a limit to the degree of cross-linking above which the biocompatibility of the hydrogel may decrease. The HA-based hydrogels are advantageous drug carriers for prolonged and controlled drug release into the targeted area [13].

**Figure 2:**
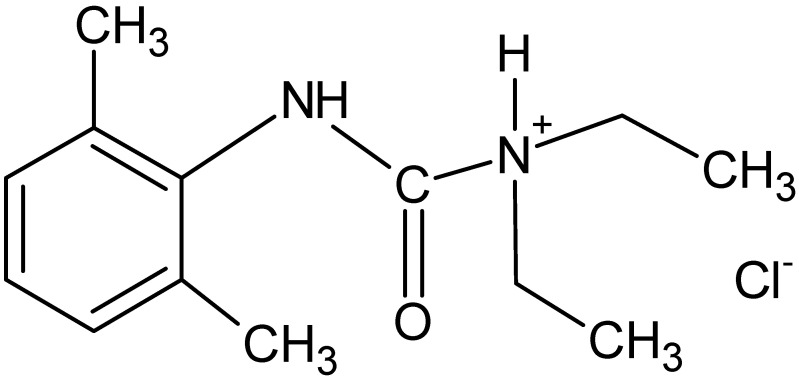
The structure of lidocaine hydrochloride.

Lidocaine hydrochloride (L) is a local anesthetic drug. Its structure includes a lipophilic aromatic ring and a hydrophilic amino group, which are linked by an amide bond (Figure 2). The amide bond is very stable. The main particle exhibits basic properties. It prolongs the drug half-life and increases the pharmacological activity of L in comparison to other local anesthetics. Apart from the local anesthetic and antiarrhythmic effects, L also has anti-inflammatory and analgesic properties. However, L also has adverse effects, mainly related to the central nervous system as well as the cardiovascular system. For this reason, the maximum recommended total dosage of L was established at 500 mg for adults and 300 mg for children; however, the maximum total dosage may need adjustment based on weight, age, and medical conditions [14].

L is used in therapy in several forms: injections, inhalations, topical gels, and transdermal patches. It is employed to relieve pain after minor surgery or invasive procedures such as biopsies, minor excisions, or dental surgery [14]. L is also used in infusion therapy against chronic pain or as an intranasal spray for trigeminal neuralgia [15,16].

**Figure 3:**
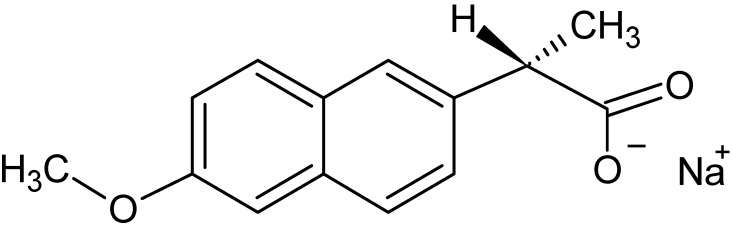
The structure of naproxen sodium.

Naproxen sodium (N) is a non-steroidal anti-inflammatory drug, i.e., a substance with analgesic, antipyretic, and anti-inflammatory properties. It belongs to the group of compounds derived from arylpropionic acid (Figure 3). The presence of two benzene rings in a condensed system enables the long pharmacological activity of this drug [17,18,19]. The asymmetric C atom results in the existence of a pair of N enantiomers. These isomers have similar physical properties. However, it was shown that the S (+) enantiomer has a higher thromboxane inhibitory activity; while the R (−) enantiomer has hepatotoxic activity; therefore, the S (+) enantiomer is used as a pharmacologically active agent [20]. The core N molecule is an acidic entity. Apart from desired therapeutic effects it may exhibit undesirable properties, e.g., an ulcerogenic effect, a negative effect on the renal system, an increased risk of cardiovascular events, and adverse skin reactions, which are controlled by maintaining therapeutic levels of N. The oral dose of N is 220 mg, while the maximum daily recommended dose is 660 mg. A dose of 440 mg of N acts for 8 to 12 h, which is the recommended dosing interval. It is the result of the relatively long half-life of N, which is 15 h [19].

The aim of the research was to evaluate the release kinetics of cationic lidocaine and anionic naproxen from HA hydrogel. The study was supplemented by an investigation of the possible interactions between the carrier and the drug by thermal, viscometric, and spectroscopic methods.

## 2. Results and Discussion

### 2.1. Viscosity Study

**Figure 4:**
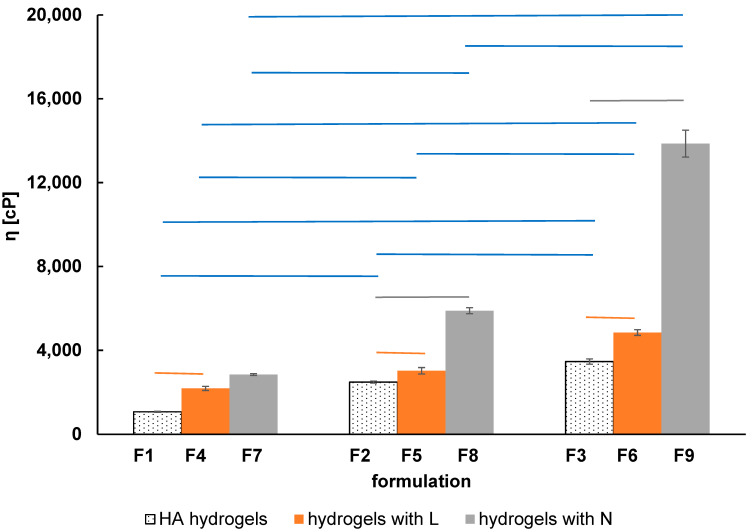
The viscosity of the formulations F1–F9 at the temperature of 37 °C. The ends of the horizontal lines show the compared formulations, that indicated statistically significant differences. The orange lines indicate the statistically significant differences arising from the L incorporation to formulations, the grey lines indicate the statistically significant differences arising from N incorporation to formulations, the blue lines indicate the statistically significant differences resulting from the various concentration of carrier in formulations; *n* = 5, *p* < 0.05.

The viscosity of formulations F1–F9 was presented in Figure 4. The composition of the obtained hydrogels is presented in Table 1.

It was noticed that the viscosity of the hydrogels increased with the concentration of the carrier, namely the viscosity increases from formulation F1 to F3, from F4 to F6, and from F7 to F9. These discrepancies arising from different HA amounts in hydrogels were marked in blue in Figure 4. This observation was in good agreement with the results presented by Kobayashi et al. [21]. It was found in this research that the increased molecular weight and concentration of HA strengthened the polymer network. The presence of functional groups in the HA chain, such as: -COOH, -OH, and -NHCOCH_3,_ facilitated the formation of hydrogen bonds. These bonds additionally stiffened the hydrogel and increased its viscosity. The statistical analysis using ANOVA with Tukey’s test gave the values of the probability *p* < 0.0001, confirming statistically significant differences between formulations F1–F3 containing different amounts of HA (3, 4, or 5 g of HA). Moreover, the statistical analysis showed that there were statistically significant differences in the viscosity of the formulations containing L, N, or drug-free. It was revealed that the lowest value of the viscosity had the formulations without drug content. However, the incorporation of both of the drugs, separately, to the hydrogels increased the viscosity. This observation may suggest the interaction between the carrier and L as well as between the carrier and N.

HA is a polyanion with accompanying cations (e.g., H^+^ or Na^+^) from the ionized carboxylic groups. Additionally, the structure of HA contains hydroxyl groups as well as acetylamino groups. According to Khunmanee et al. [22], these three groups are good sites for the bonding of various molecules. Lidocaine hydrochloride (Figure 2) is a cation associated with a chloride anion. The ionized carboxyl group of the HA molecule may associate with the lidocaine cation [23] This reaction may explain the increase in viscosity of formulations F4–F6 in comparison to formulations F1–F3. Naproxen sodium was present in a solution as an anion associated with the cation Na^+^. In the case of formulations F7–F9, the increase in viscosity in comparison to the viscosity of formulations F1–F3 was a result of the reaction of the -OH group from HA with the ionized group of -COO^−^ coming from naproxen sodium to form an ester bond, as it was proposed by Larrañeta et al. [24].

### 2.2. Release Study

**Figure 5:**
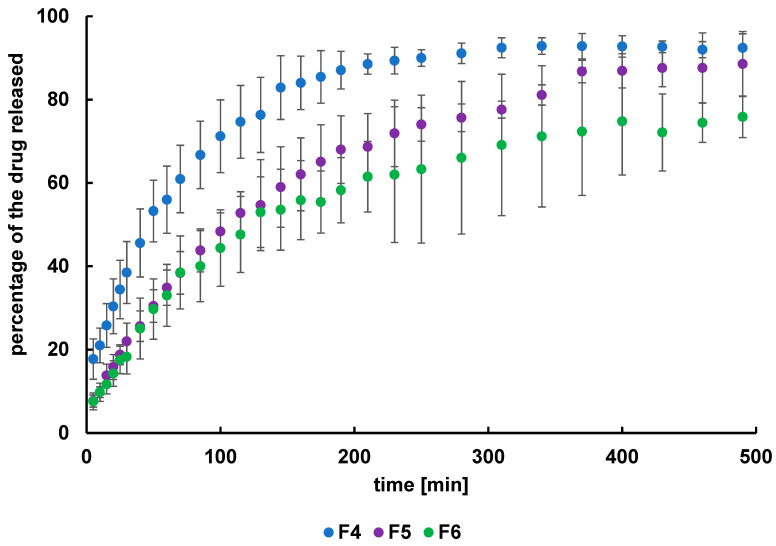
The release curves of L from formulations F4–F6.

The obtained dissolution profiles of L from the formulations F4–F6 were presented in Figure 5. The highest amount of the drug released to the acceptor fluid (92.4 ± 3.4%) was recorded from formulation F4, which had the lowest concentration of HA as well as the lowest viscosity. In the case of the dissolution of L from formulations F5 and F6, initially, the concentrations of the L in the medium were similar, but after about 100 min, the amount of the active substance released was higher from F5 than from F6. It may be a result of the drug’s transportation at the beginning from the surface and in the second stage from the interior of the hydrogel to the medium. Finally, the amount of L released from F5 was lower than that from F4 and was 88.6 ± 8.0%, although it was higher than the amount released from F6 (75.9 ± 5.0%).

**Figure 6:**
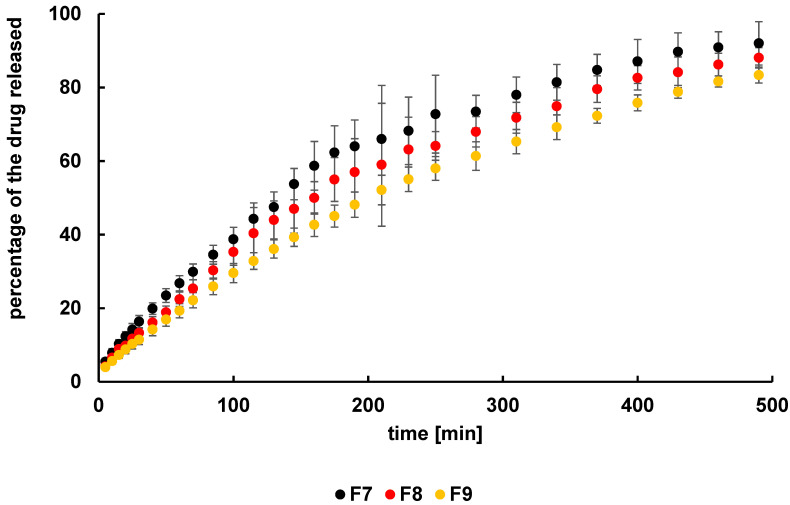
The release curves of N from formulations F7–F9.

The dissolution curves of N from hydrogels F7–F9 were presented in Figure 6. It was found that the amount of the substance released depended on the carrier concentration and thus the viscosity of the hydrogel, similarly to the release of L from formulations F4–F7. The tendency was that the highest percentage of the drug released was from the F7 formulation (92.0 ± 5.9%), which contained the lowest concentration of HA and had the lowest viscosity; however, the highest amount of N was released from the F9 hydrogel (83.4 ± 2.2%), which had the highest concentration of HA and the highest viscosity. It may be proposed that the polymer net—reinforced with additional internal hydrogen bonds between the functional groups of HA—constituted a steric hindrance and reduced the transport of the drug to the acceptor fluid. This is consistent with the study by Briggs et al. [25]. It was revealed in this study that the drug release rate from hydrogels depended on the concentration as well as the crosslinking density of the polymer.

Moreover, there exists a saturation point in the ability to control drug release rates through a combination of chemical and physical crosslinkers. The obtained results indicated that various properties of hydrogel preparations influenced the amount of L and N released. Such information is very important in the design of the controlled-release polymer drug carriers that will deliver the desired dose of the drug substance within a defined period of time [25].

### 2.3. Release Curves Comparison

The release curves were compared by calculating the difference factor f_1_ and the similarity factor f_2_. The obtained values for these factors are listed in Table 2. It was noticed that the release curves of L for F5 and F6 were similar; the value of f_1_ was below 15 and the value of f_2_ was above 50, and according to FDA recommendations, they can be considered the same [26]. The comparison of the release profiles of L from F4 and F5, as well as from F4 and F6, indicated the differences, and the obtained values of f_1_ and f_2_ presented in Table 1 were marked bold. The discrepancies may arise from the variable amount of HA in these hydrogels. However, the difference between the release curves F5 and F6 was not found, reflecting that the disparity of the HA level in these preparations was insufficient to influence the drug transport. In the case of the dissolution profiles of N from F7–F9, the difference was found only between the release curves F7 and F9. The respective parameters in Table 1 were marked in bold. The difference may be attributed to the higher HA concentration in formulations F7 and F9. The differences in carrier amount between the F7 and F8 hydrogels and between the F8 and F9 formulations were equal, although the difference between F7 and F9 was higher, explaining the obtained results.

The differences between the release curves were also analyzed using statistical methods. The values of parameters a and b delivered from Equation (9) were collected in Table 3. The comparison of these parameters based on the z-test indicated the statistically significant differences between profiles F4–F5, F4–F6, F5–F6, F7–F9, and F8–F9. Only between the curves F7–F8 the discrepancy was not found. This analysis confirmed the dissimilarities revealed on the basis of the f_1_ and f_2_ coefficients. However, these factors do not indicate the differences between F5–F6 and F8–F9, meaning that the inconsistency was not high enough. Both methods do not indicate the difference between the release plots of F7 and F8.

It can be concluded that the HA level affects the release of L or N from the tested hydrogels, although, in some cases, the differences in polymer concentration were insufficient to indicate any effect of the carrier concentration on the drug release.

### 2.4. Kinetic Analysis

**Figure 7:**
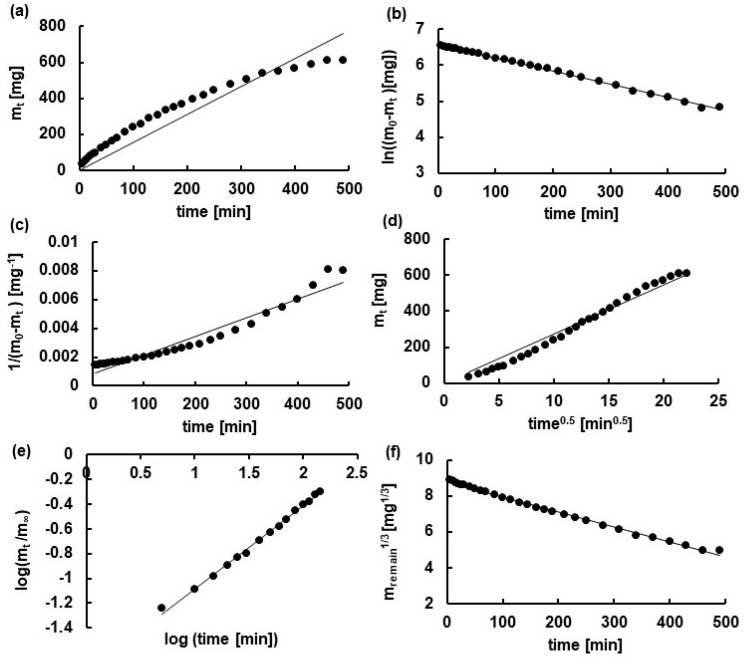
The theoretical plots (lines) fitted to the experimental data (points) obtained for the release of N from formulation F9; (**a**) zero-order model, (**b**) first-order model; (**c**) second-order model; (**d**) Higuchi model; (**e**) Korsmeyer-Peppas model; (**f**) Hixon-Crowell model.

The release curves were analyzed using zero-, first-, and second-order kinetics as well as Higuchi, Korsmeyer-Peppas, and Hixon-Crowell equations [27]. The example of the fitting of the experimental data coming from the release of N from F9 to the equations was presented in Figure 7.

Based on the least squares method, the values of the release rate constants (k), the half release time (t_0.5_), the correlation coefficient (R^2^) employing different kinetic equations, as well as the n parameter from the Korsmeyer-Peppas model, were calculated and are listed in Table 4. The values were presented according to Joint Committee for Guides in Metrology (JCGM) recommendations [28].

The highest value of the correlation coefficient R^2^ indicated the most adequate model describing the investigated process. The Korsmeyer-Peppas model (7) was the equation that best characterized the dissolution of both drugs in the studied formulations. In this model, very high values of the correlation coefficients were obtained (from 0.96 ± 0.01 to 0.99 ± 0.01). 

Generally, the Korsmeyer-Peppas Equation (7) may be used when the dissolution occurs in a one-dimensional way and the system width/thickness or length/thickness relation is at least 10 [29]. This expression is employed to describe the dissolution of pharmaceutical polymeric dosage forms if the dissolution mechanism is unknown or when more than one type of drug transport is included.

Peppas [30] in his work proposed the n value in Equation (7) to characterize the transport of the drug from polymeric films to the acceptor fluid. He concluded that for values of *n* = 0.5, the mass transport follows Fick’s diffusion, although for values higher than 0.5, the drug transfer follows a non-Fickian model, namely, between 0.5 and 1.0, it is anomalous transport, for *n* = 1.0, it is case-II transports, meaning the zero-order model, and for n higher than 1.0, it is super case-II transport. Moreover, Ritger and Peppas [31] showed that in the case of Fickian diffusion, the values of the parameter *n* are 0.50; 0.45; 0.43 for the drug transfer from slabs, cylinders, and spheres, respectively. For tablets, it can be between 0.43 and 0.5. When the drug is released from cylinders, the value of n is 0.89 instead of 1.0 [29,32,33,34].

In the present work the values of the parameter n describing the transport of the drug from the carrier to the acceptor fluid were in all cases in the range of 0.56 ± 0.04 to 0.72 ± 0.04, indicating that the transport did not occur according to Fick’s law. The release of L as well as N was controlled by both the diffusion of the active substance and the relaxation of the carrier chains [35]. It is worth noting that the value of n slightly increased with the increase in HA concentration. It is consistent with the results obtained by Khan et al. [36]. Researchers postulated in this work that the increased carrier concentration changed the way of the drug’s transportation from anomalous transport (*n* in the range of 0.5–1.0) to case-II transport (*n* = 1). In the microscopic analysis, it was revealed that there was a relaxation response of the polymer chain caused by the presence of the solvent. The molecular volume of the hydrated polymer increased, reducing the free space coming from the presence of micropores. This led to a shift in the drug release mechanism.

In the case of the dissolution of L, the value of k_K-P_ derived from Korsmeyer Peppas model was the highest when the drug was released from formulation F4. It was (6.69 ± 0.84) × 10^−2^ min^−0.56^, and confirmed that L was released the fastest from formulation F4 with the lowest concentration of the carrier. The analysis of the release of L from F5 and F6 gave very similar values for the rate constants: (2.63 ± 0.88) × 10^−2^ min^−0.65^ and (2.97 ± 0.70) × 10^−2^ min^−0.64^, respectively, although the values of the half release time increased with the HA concentration (from F4 to F6). The release rate constants k_K-P_ obtained when N was released from F7–F9 decreased slightly with the HA amount increasing in hydrogels. The values of the half release time were similar for the release of N from formulations F7 and F8, and both were lower than those from the hydrogel F9, which contained the highest concentration of the polymer. It was consistent with the results obtained from the release of L.

The analysis of the release of L from the F4–F6 formulations based on zero-order kinetics was possible only for the time range from the beginning of the test to 40 min. The values of kinetic parameters are collected in Table 4 and refer to the initial 40 min of the experiment. It was not possible to fit the zero-order equation to the entire L-release profile, and we assumed that the zero-order kinetics was not an appropriate model to describe the studied processes of L release. However, the whole release profile of N was fit to the zero-order model. The obtained kinetic parameters from this equation were very close, but the values of R^2^ were not very high (from 0.71 ± 0.05 to 0.90 ± 0.03). The obtained R^2^ suggested low adherence of the zero-order model to the studied processes, and the kinetic parameters should not be taken into consideration.

The zero-order kinetics (3) are usually used to describe the release of the drug from transdermal systems, matrix tablets with low soluble drugs, coated forms, and osmotic systems. In this model, the same amount of the drug is released per unit of time, meaning that the rate of dissolution is constant [37,38]. However, although the zero-order model is often used to describe the drug release from hydrophilic gels, in the presented work this expression was unsuitable, in particular to describe the release of L.

The kinetic study of the release of L from F4–F6 has shown that the values of k_1_ and k_2_ decreased with the increase in the HA concentration. However, when N was released from the hydrogels, the values of k_1_ and k_2_ obtained for F7 and F8 were similar, although they were higher than those derived for the F9 formulation, suggesting that N was released faster when the amount of the polymer in the hydrogel was lower.

In the first-order model (4), the rate of the process is proportional to the concentration of the drug. This relationship can be used to characterize absorption and/or elimination of the drug [39,40]. Additionally, it may be used to describe the early dissolution of a poorly water-soluble drug in a water-soluble matrix. In the second-order model (5), the rate of the process is proportional to the square of the concentration. In nature, most processes follow first-order kinetics, so it is most often used in data analysis. In this work, the first-order and second-order kinetics equations were also employed. The obtained R^2^ values ranged from 0.83 ± 0.04 to 0.99 ± 0.03 and from 0.77 ± 0.20 to 0.91 ± 0.02 for the first- and second-order models, respectively. Gouda et al. [34] obtained from the first-order analysis the values of R^2^ of 0.83 and 0.92, establishing that the studied process did not follow the first-order expression. It can be concluded that in this work, the dissolution of N from F9 followed the first-order equation, but the release of the drug from F5, F7, and F8 did not follow this model perfectly. However, this expression was inadequate to describe the drug release from F4 and F6. All the studied systems did not follow second-order kinetics.

The comparison of the values of k_H_ obtained from the release of L revealed that the k_H_ value of the drug dissolution from F4 was lower than from F5 and F6, which were close. However, the value of R^2^ for the L release from F4 using the Higuchi model was rather low (0.67 ± 0.16), and this may be the reason for the low calculated value of k_H_. It should be mentioned that the values of k_H_ obtained for the N release from hydrogels studied slightly decreased with the increase of the HA concentration (from F7 to F9). Similar results were obtained in the case of the Hixon-Crowell model. The values of the release rate constants for the dissolution of L from F4–F6 were lowest in the case of F6. The values of k_H-C_ for the release of N from F7 and F8 were very close, although the value of k_H-C_ obtained from the N dissolution in F9 was lower. 

The expression proposed by Higuchi (6) characterizes the Fickian diffusion-based process. This equation can be applied to describe the drug release from several types of modified release pharmaceutical dosage forms, such as transdermal systems, and matrix tablets with water soluble drugs [29]. In the presented study, transdermal systems were investigated. However, the Higuchi model was inappropriate to describe the drug dissolution, and the analysis indicated that the release did not follow this expression perfectly. The values of the parameter R^2^ were in the range of 0.67 ± 0.16 to 0.96 ± 0.01. These results suggested that the drug release did not follow diffusion according to Fick’s law.

The Hixon-Crowell model (8) contains the constant k_H-C_ that represents the surface-volume relation. This expression may be applied to the dissolution of the drug from tablets or another system in which the drug release takes place in the area that is parallel to the drug surface if the tablet sizes decrease proportionally in such a way that the initial geometrical shape stays constant all the time [33]. In the present investigation, the calculated values of R^2^ were in the range of 0.79 ± 0.05 to 0.99 ± 0.03, meaning that only in the case of the drug release from F9 was this model appropriate. 

Obviously, the concentration of the carrier influenced the drug release. It was consistent with the results presented in our previous work [41] as well as with the data obtained by Khan et al. [36], Szcześniak et al. [42], and Liu et al. [43]. The increase in polymer concentration impeded the rate of drug dissolution, which may lead to a change in the drug release mechanism.

### 2.5. FTIR Study

**Figure 8:**
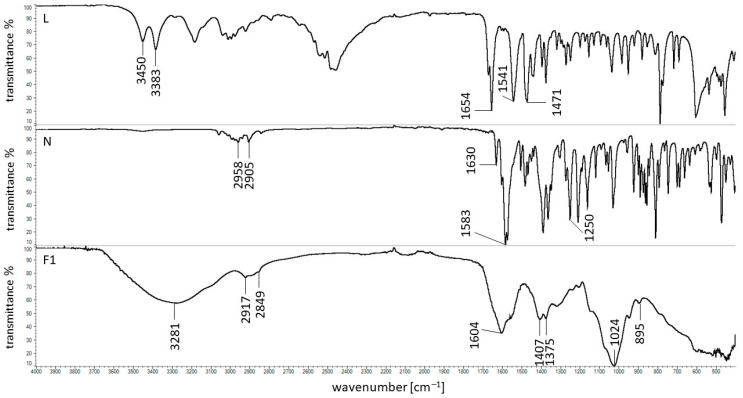
FTIR spectra of L, N and formulation F1.

The spectrum of lidocaine hydrochloride (L) is presented in Figure 8. The observed maxima were in good agreement with the signals obtained by Abu-Huwaij et al. [44], Wei et al. [45], and Li et al. [46]. The bands at 3450 cm^−1^ and 3383 cm^−1^ were attributed to the stretching of the N-H group. The sharp peak at 1654 cm^−1^ corresponded to the stretching of the carbonyl group C=O in the amide group. The intensive maxima at 1541 cm^−1^ and 1471 cm^−1^ were assigned to C-N stretching, with the band at 1541 cm^−1^ corresponding to amide vibrations.

The spectrum of naproxen sodium (N) was also shown in Figure 8. It was consistent with the spectrum recorded by Sharma et al. [47] and Jamrógiewicz et al. [48]. The characteristic maxima of the functional groups were found at the wavenumbers 2958 cm^−1^ and 2905 cm^−1^, which were related to the C-H group. The signal at 1630 cm^−1^ was responsible for the stretching of the aromatic group C-C coming from the acid. The band at 1583 cm^−1^ was attributed to the stretching of the COO^-^ group, and the signal at 1250 cm^−1^ belonged to the stretching of the C-O group coming from the acid.

The FTIR spectrum of HA was presented in our previous work [41] and was consistent with the spectra obtained by Reddy et al. [49] and Akkarad et al. [50]. In Figure 8, as an example, the spectra of the F1 formulation composed only of HA and water were shown. The spectra of F2 and F3, which contain the same composition, were similar. The wide band at 3281 cm^−1^ was assigned to the stretching of the N-H group and O-H group. The signals at 2917 cm^−1^ and 2849 cm^−1^ were related to the stretching of the C-H group. The maximum at 1604 cm^−1^ was attributed to the amid II group, and the band at 1407 cm^−1^ corresponded to the C-O group with the C=O combination. The maximum at 1743 cm^−1^ indicating the presence of the -COOH group, was not observed, confirming the existence of the deprotonated form of HA. The band at 1375 cm^−1^ was assigned to the presence of CH, CH_3_, C-O-H groups. The sharp peak at 1024 cm^−1^ indicated the stretching of the C-O-C, C-O, and C-O-H groups. The maximum at 895 cm^−1^ means the presence of the C-O-C group and the deformation of the carbonyl and hydroxyl groups. It was noticed that all the characteristic bands of HA were present on the spectrum of F1. The possible hydrogen bonds between the deprotonated carboxyl groups of HA and water molecules may occur, although the wide signal at 3281 cm^−1^ overlapped with the band coming from hydrogen bonding, which is usually present in the spectrum in the range of 2500–3300 cm^−1^. Additionally, hydrogen bonds between the polysaccharide chains may also occur, according to the literature [24].

**Figure 9:**
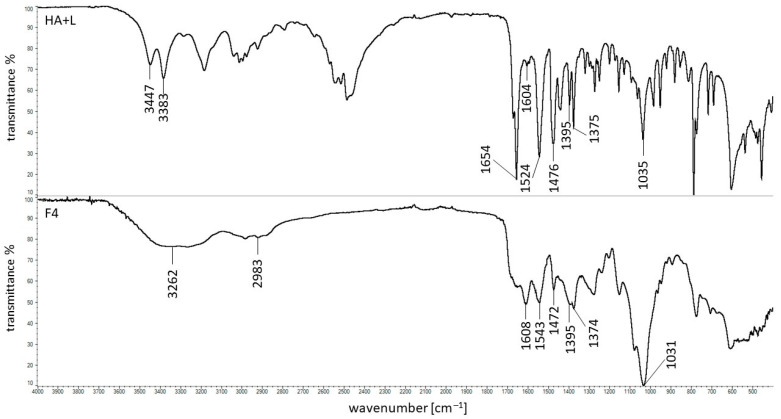
FTIR spectra of the physical mixture of HA + L and the formulation F4.

The FTIR spectrum of the physical mixture of HA + L as well as the spectrum of the formulation F4 were presented in Figure 9.

It was found that all the characteristic bands of L and HA were present on the spectrum of the physical mixture of L + HA, which confirmed the lack of interaction between these components. However, in the case of the spectrum of the F4 formulation, composed of L, HA, and water, the lack of maximums at 3450 cm^−1^, 3383 cm^−1^ and 1654 cm^−1^ belonging to L and coming from N-H and C=O groups was noticed. The signals assigned to the presence of HA were found, although the bands at 1395 cm^−1^ and 1375 cm^−1^ changed the intensity. These observations were also found in the spectra of formulations F5, and F6, which contained the same ingredients but different amounts of HA. It may suggest that there was an interaction between the C-O group from HA and the amino group from L. This was consistent with the results obtained by Mrestani et al. [23]. According to this research, the weak interaction between the glucuronic acid (GluA) from HA and L was revealed.

**Figure 10:**
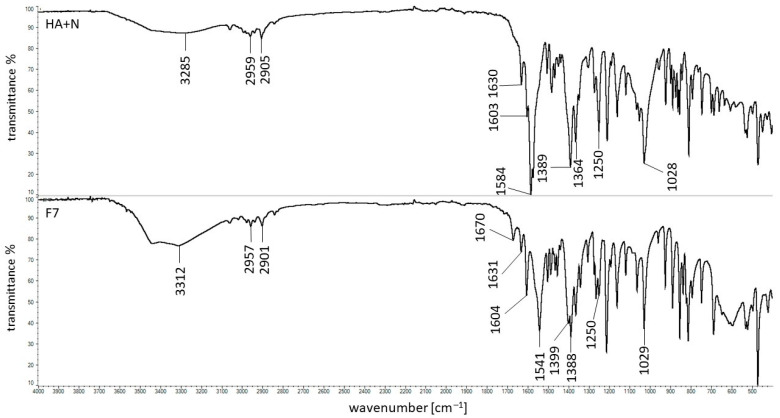
FTIR spectra of the physical mixture of HA + N and the formulation F7.

The FTIR spectrum of the physical mixture of HA + N as well as the spectrum of the formulation F7 composed of HA, N, and water were presented in Figure 10.

All the characteristic bands assigned to HA and N were present in the spectrum of the physical mixture of these ingredients, indicating the lack of interaction between these compounds. However, in the spectrum of the formulation F7 composed of HA, N, and water, the maximum of N at the wavenumber 1583 cm^−1^ assigned to the presence of COO^-^ group was shifted towards 1541 cm^−1^. The broad signal of HA at 3285 cm^−1^ was shifted to 3312 cm^−1^ by changing its intensity and shape. The frequency range between 3100 cm^−1^ and 3600 cm^−1^ was the hydroxyl vibration region [51]. The maximum at 1407 cm^−1^ attributed to the C-O with C=O deformation group was shifted to 1399 cm^−1^ and the weak band at the frequency of 1375 cm^−1^ corresponded to C-O-H deformation was not visible [50]. Additionally, the new signal at 1670 cm^−1^ appeared. The spectra of F8 and F9 were similar. All the noticed discrepancies may suggest the interaction between the HA chain and the N molecule. Similar results were observed by Larrañeta et al. [24] in the FTIR study of HA and Gantrez S97 polymers. It was postulated that an ester bond would form between the hydroxyl group from the HA chain and the carboxyl group from the Gantrez S97 polymer. It was also consistent with the results obtained in viscometric tests included in Section 2.1 of this paper.

### 2.6. DSC Investigations

**Figure 11:**
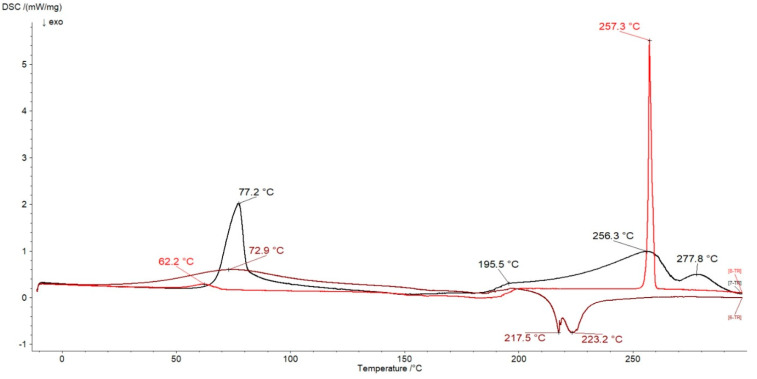
The thermograms of L (black line), N (red line) and F1 (brown line).

The thermograms of L, N, and formulation F1 were shown in Figure 11. On the DSC curve of L, the sharp endotherm at a temperature of 77.2 °C was present and can be assigned to the melting point of the compound [45,52]. The peaks over 200 °C were related to the degradation of the molecules [53].

The thermogram of N showed a weak peak at 62.2 °C connected with the water evaporation, and the second sharp maximum at 257.3 °C corresponded to the melting point of the drug [52]. The thermogram of pure HA was presented in our previous work, and the signals at 69.0 °C, 124.2 °C, 228.5 °C, and 246.7 °C were observed [41]. In Figure 11, the thermogram of the formulation F1, composed of HA and water, was also presented. It was found that the characteristic endotherm of pure HA at 69.0 °C was shifted to 72.9 °C on the curve of F1. The exotherms of HA at 228.5 °C and at 246.7 °C were found at 217.5 °C and at 223.2 °C on the F1 curve, respectively. The weak endotherm of HA at 124.2 °C connected with the water evaporation was not visible on the thermogram of F1. The thermograms of F2 and F3 were similar to the DSC curve of the F1 formulation.

**Figure 12:**
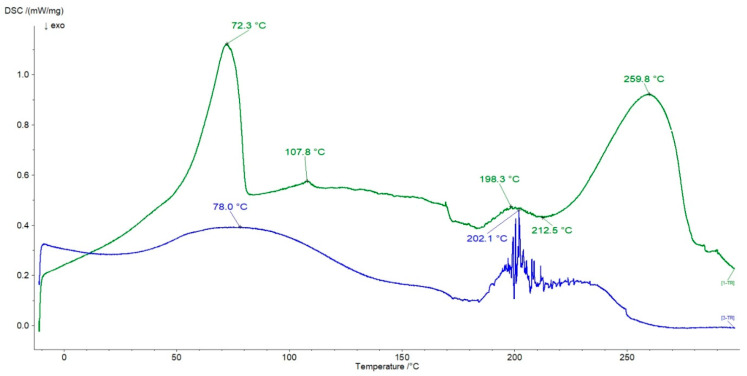
The thermograms of the physical mixture of HA + L (green line) and the formulation F4 (blue line).

The thermogram of the physical mixture of HA + L and the formulation F4 were depicted in Figure 12.

The peaks of L were present on the thermogram of the physical mixture HA + L at 72.3 °C, 198.3 °C, and 259.8 °C. The peaks at 107.8 °C and 212.5 °C were related to HA. The endotherm at 72.3 °C apart from L may also belong to HA. However, the exotherm of HA at 246.7 °C was not observed on the curve of the physical mixture. It may be overlapped by the peak at 259.8 °C coming from the strong endotherm of L. The obtained observation may suggest no interaction between the components of the physical mixture of HA + L. The thermogram of the formulation F4 was very interesting. At 78.0 °C, the very wide endotherm assigned to HA and L was found. Between 195.0 °C and 220.0 °C, there was a wide signal composed of several minima and maxima, which were difficult to clearly assign to HA or L. The thermograms of formulations F5 and F6 were similar to the plot of formulation F4. The changes on the thermogram of F4 compared with the DSC curve of the physical mixture of HA + L and pure ingredients may indicate the interaction between HA and L molecules. According to Mrestani’s evaluation of L-HA interaction, there is the possibility of complexation between the carboxyl HA group and the ionized nitrogen of L [23], which may lead to the observed difference between the physical mixture thermogram and the formulation thermogram. The observation corresponded well with the results obtained from the FTIR study.

**Figure 13:**
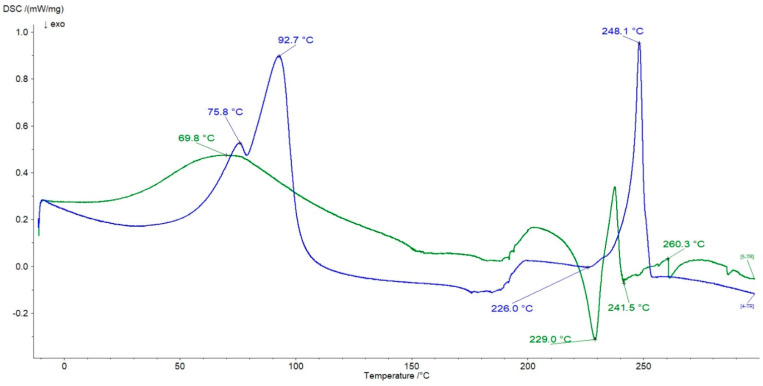
The thermograms of the physical mixture of HA + N (green line) and the formulation F7 (blue line).

In Figure 13 the thermograms of the physical mixture of HA + N and the formulation F7 were illustrated.

The peaks belonging to N were present on the curve of the physical mixture of HA + N at 69.8 °C and 260.3 °C, respectively. However, the signal at 69.8 °C also overlapped with the endotherm assigned to HA. Both exotherms of HA at 228.5 °C and 246.7 °C were present on the curve of the physical mixture of HA + N at 229.0 °C and 241.5 °C, respectively. These observations allowed us to conclude that there was no interaction between HA and N in the physical mixture of these ingredients. In the case of the thermogram of the formulation F7, the peak at 75.8 °C corresponding to the evaporation of water can be assigned to HA as well as to N. The endotherm assigned to the melting point of N was observed at 248.1 °C. The weak exotherm at 226.0 °C belonging to HA was noticed, although the second exotherm of HA at 246.7 °C was not found. Moreover, a new endotherm at 92.7 °C appeared on the DSC curve of the formulation F7. The thermograms of formulations F8 and F9 were similar to the curve of F7. The lack of the second endotherm of HA at 246.7 °C and the presence of the new peak at 92.7 °C on the F7 curve may indicate bond formation between HA and N and is consistent with the results obtained in FTIR studies. The resulting double peak (75.8 °C and 92.7 °C) in the formulation F7 thermogram may also suggest the interaction between the carboxyl groups of N and the hydroxyl groups of HA, as supported by the hypothesis of Larrañeta et al. [24], who evaluated the crosslinking process of HA, performed with an acidic copolymer of methylvinylether and maleic anhydride. However, the variability of melting points may result from the formation of other products [54].

The formation of bonds between molecules is very important in pharmaceutical science and medicine. According to Kopecek et al. [55], the possibility of an interaction between the carrier and another molecule may influence the pharmacokinetics, biorecognition, distribution, and subcellular location as well as the therapeutic efficacy. In the case of polymers that are degraded only by hydrolysis, the bond formation with a drug may reduce the degradation of the resulting system. The polymer-drug conjugates may have different architectures, such as linear, star, graft, or branched copolymers, that have an impact on the rate of drug release, IC50 doses, biodistribution, and in vivo efficacy. It was revealed that the star conjugate showed higher tumor growth inhibitory activity than the linear one [56]. However, Širová et al. [57] found that the linear conjugates increased the treatment effects and the star conjugate reduced the treatment effectiveness. The interaction between molecules is also important for the penetration of a drug-bound polymer through cell membranes. The attachment of cell-specific ligands enables active targeting of conjugates. As a result, the biorecognition of such systems increases. After attaching such moieties, the resulting complex is more easily directed to the appropriate cell receptor, followed by transport into the cell interior, and the ligand is detached [55].

## 3. Materials and Methods

HA was obtained from Esent (Szczecin, Poland), and L was bought at Sigma-Aldrich (Steinhelm, Germany). N was a gift from Hasco Lek (Wrocław, Poland). Sodium hydroxide and potassium dihydrogen phosphate were derived from Chempur (Piekary Śląskie, Poland). The phosphate buffer, pH = 7.5, was prepared according to the European Pharmacopoeia [58]. The cellulose membranes were obtained from Carl Roth (Karlsruhe, Germany).

### 3.1. Hydrogels Preparation

The appropriate amount of high molecular weight HA (1.1 MDa) was combined with water to form a hydrophilic gel. The formulation was mixed with a homogenizer (Unidrive X 1000D, Cat, Staufen, Germany) to obtain a homogeneous form. The obtained preparation was left for 24 h at a temperature of 6 °C. The compositions containing a drug were prepared in a similar way. The required amount of the drug was dissolved in water, combined with an appropriate amount of HA, and homogenized. The formulation was deaerated for 24 h at 6 °C. The composition of the obtained hydrogels is presented in Table 1.

The dose of lidocaine used for the topical anesthesia in the cataract surgery was 2% [59]. 2% gel of lidocaine was also proposed as the topical anesthetic agent for phacoemulsification surgery [60]. In the presented study, the concentration of L in formulations F4–F6 was also 2%. The N concentration in the formulations F7–F9 was 10%, reflecting the anti-inflammatory and analgesic concentrations in therapeutic hydrogels with N.

### 3.2. Viscosity Study

The viscosity of the obtained hydrogels was evaluated in a rotational viscometer (DV2T, Brookfield, Middleboro, MA, USA). The formulations were placed in a water bath (W215C, Laboplay, Bytom, Poland) and heated to a temperature of 37 °C. The tests were carried out for 1 min with a spindle rotation speed of 200 rpm using dedicated spindles with a number from 4 to 7, with five repetitions to enable calculation of the average value with the standard deviation (SD). The value of the measured dynamic viscosity, i.e., the dynamic viscosity coefficient, was displayed directly on the display panel.

### 3.3. Release Study

The drug release study from the hydrogels was carried out in a dedicated bath (ERWEKA, DT 126 Light, Heusenstamm, Germany) with extraction cells with cellulose membranes, using the pharmacopoeial paddle over the disc method [61]. The preparation was introduced in the extraction cell that was subsequently placed in the acceptor fluid, i.e., 1 L of phosphate buffer of pH = 7.5 at 37 °C and at the rotation speed of the paddles at 50 rpm. The samples of 3 mL were taken at defined time intervals and replenished with fresh acceptor fluid. Six tests were performed for each formulation. The amount of the drug released was determined spectrophotometrically (JASCO V-530, Tokyo, Japan) by reading the absorbance of L at a wavelength of 262 nm and the absorbance of N at a wavelength of 271 nm. The wavelengths were selected according to the spectra of L and N in the phosphate buffer, pH = 7.5. The concentration of the drug released was calculated from the absorbance-concentration calibration curves prepared at the appropriate wavelength.

### 3.4. Difference Factor and Similarity Factor

The comparison between the release curves was conducted by calculating the difference factor (f_1_) and the similarity factor (f_2_) recommended by the FDA [26] and employing the following equations:

(1) $$f_{1}=\frac{\sum_{t=1}^{n}\left|R_{t}-T_{t}\right|}{\sum_{t=1}^{n}R_{t}}\times 100$$

(2) $$f_{2}=50\times log\left\{{\left[1+\frac{\sum_{t=1}^{n}{\left(R_{t}-T_{t}\right)}^{2}}{n}\right]}^{-0.5}\times 100\right\}$$

where n—the number of time points, R_t_—the dissolution value of the reference batch at time t, and T_t_—the dissolution value of the test batch at time t.

### 3.5. Kinetic Study

The obtained release profiles of L and N were analyzed employing zero-, first-, and second-order kinetics as well as Higuchi, Korsmeyer-Peppas, and Hixon-Crowell models [27]. According to Korsmeyer et al. [62], the first 60% of the drug released fit into this model. On the basis of the aforementioned equations, the release half-time and release rate constants were determined. The highest value of the correlation coefficient R^2^ indicated the best model that characterized the process studied. The used equations were presented in our previous works [63,64,65] and are as follows:

(3) $$\mathrm{Zero-order}\;(\mathrm{Z-O})\;m_{t}=m_{b}+k_{0}t$$

(4) $$\mathrm{First-Order}\;(\mathrm{F-O})\;\ln\left(m_{0}-m_{t}\right)=\ln\left(m_{0}\right)-k_{1}t$$

(5) $$\mathrm{Second-Order}\;(\mathrm{S-O})\;\frac{1}{\left(m_{0}-m_{t}\right)}=\frac{1}{m_{0}}-k_{2}t$$

(6) $$\mathrm{Higuchi}\;(H)\;m_{t}=k_{H}t^{0.5}$$

(7) $$\mathrm{Korsmeyer-Peppas}\;(\mathrm{K-P})\;\log\left(\frac{m_{t}}{m_{\infty }}\right)=\log k_{K-P}+n\log t$$

(8) $$\mathrm{Hixon-Crowell}\;(\mathrm{H-C})\;{m_{0}}^{\frac{1}{3}}-{m_{r}}^{\frac{1}{3}}=k_{H-C}t$$

where m_t_—the amount of the drug released in time t; m_b_—the amount of the drug in the solution before the release, usually it is 0; k_0_—the zero-order release rate constant; m_0_—the amount of the drug in the formulation before the dissolution; k_1_—the first-order release rate constant; k_2_—the second order release rate constant; k_H_—the Higuchi rate constant; m_∞_—the amount of the drug released after infinitive time, k_K-P_—the Korsmeyer-Peppas rate constant, n—the parameter indicative of the drug release mechanism; m_r_- the amount of the drug remaining in the formulation in time t; k_H-C_—the Hixon-Crowell rate constant.

### 3.6. Statistical Analysis

The statistical study was performed with the computer program Statistica 13 [66]. On the basis of five viscosity measurements, the mean value of the viscosity of each formulation with the standard deviation (SD) was determined. The Shapiro-Wilk test at the significance level of 0.05 was used to check whether the samples came from a population with a normal distribution. The homogeneity of the variance was checked with the Brown-Forsaythe test. The analysis of variance (ANOVA) was performed at a significance level of 0.05. A post-hoc test—Tukey’s test—was used to identify the groups responsible for the occurrence of the statistically significant differences.

The drug release curves, representing the percentage of drug released vs. time, were obtained by determining the mass released at each time point from the six series of tests, and then calculating the mean percentage released with the standard deviation (SD). The obtained experimental points were fitted to Equation (9) as follows:

(9) $$\%\mathrm{of}\;\mathrm{the}\;\mathrm{drug}\;\mathrm{released}=\frac{a\times t}{b+t}$$

(10) $$\underset{t\to \infty }{\lim}(\%\mathrm{of}\;\mathrm{the}\;\mathrm{drug}\;\mathrm{released})=a$$

(11) $$\%\mathrm{of}\;\mathrm{the}\;\mathrm{drug}\;\mathrm{released}=\frac{a}{2}$$

Indicating that $\frac{a}{2}$ corresponded b, t means time. Based on the non-linear estimation, the parameters a and b were determined for each release profile. The comparison between the obtained parameters a and b was carried out using the z-test at a confidence level of 95%.

The kinetic study was performed employing the least squares method at a 95% confidence level. The kinetic parameters were calculated for each of the six release curves, and then the mean values with standard deviation were attained.

### 3.7. FTIR Measurements

The FTIR (Fourier Transform Infrared) study was conducted employing the FTIR spectrometer with ATR mode (Nicolet iS50, Thermo Scientific, Waltham, MA, USA). Formulations F1–F9 were dried at a temperature of 6 °C and were ground in a mortar. Physical mixtures of powders of HA + L and HA + N were also grounded. Spectra of all formulations, physical mixtures, and pure components were recorded in the wavenumber range from 4000 to 400 cm^−1^. 32 scans of each sample were collected at a speed of 65 scans per 1 min at a resolution of 16 cm^−1^.

### 3.8. DSC Measurements

The thermal study of formulations F1–F9, physical mixtures (HA + L and HA + N), as well as pure components, was performed using the DSC (Differential Scanning Calorimeter) calorimeter (214 Polyma, Netzsch, Wittelsbacherstraße, Germany). The procedure for sample preparation was the same as in the case of FTIR measurements. The sample of 3–5 mg was put into an aluminum crucible, closed with a lid, pressed, placed in the apparatus, and studied in the temperature range of −10–300 °C under a nitrogen atmosphere with a flow rate of 25 mL/min.

## 4. Conclusions

According to the viscometric tests of the obtained formulations, the increase in the concentration of the carrier results in an increase in the viscosity of the obtained HA hydrogel. The introduction of lidocaine hydrochloride or sodium naproxen into the HA hydrogel also leads to an increase in the viscosity of the preparation. The release studies of N or L have shown that the amount of the drug released within 8 h decreases with an increase in the carrier concentration. The increase in the polymer concentration also affects the course of drug release curves. In addition, kinetic analysis showed that the release of drugs from the obtained hydrogels was best described by the Korsmeyer-Peppas model. According to this model, the drugs are released the fastest from formulations with the lowest concentration of HA. The parameters n indicated that both drugs were transported from the hydrogels to the acceptor fluid via the anomalous, non-Fickian, diffusion transport mechanism. The FTIR study and DSC investigations revealed that bond formation occurred between HA and L as well as between HA and N. It was consistent with the results obtained from the viscometric study. The performed research showed that the polyanionic HA carrier may form a bond with the cationic L as well as with the anionic N.

## Figures and Tables

**Table 1:** The composition of the obtained hydrogels.

Formulation	F1	F2	F3	F4	F5	F6	F7	F8	F9
HA [g]	3	4	5	3	4	5	3	4	5
Water [g]	197	196	195	193	192	191	177	176	175
L [g]	-	-	-	4	4	4	-	-	-
N [g]	-	-	-	-	-	-	20	20	20

**Table 2:** The calculated values of the difference factor and the similarity factor.

f_1_					
Formulation	F5	F6	Formulation	F8	F9
F4	**22.86**	**46.71**	F7	1.89	**21.40**
F5	-	13.19	F8	-	12.37
f_2_					
formulation	F5	F6	formulation	F8	F9
F4	**38.32**	**32.09**	F7	89.89	**48.52**
F5	-	55.01	F8	-	61.93

**Table 3:** The obtained values of parameters a and b from the Equation (9).

Formulation	F4	F5	F6	F7	F8	F9
a	105.5 ± 1.3	113.7 ± 1.4	91.5 ± 1.5	136.2 ± 4.6	134.2 ± 6.5	154.2 ± 3.1
b	47.8 ± 2.3	133.4 ± 4.2	105.7 ± 4.9	222.3 ± 16.1	203.7 ± 21.5	416.4 ± 14.3

**Table 4:** The calculated kinetic parameters derived from the release of L and N from formulations F4–F9.

Kinetic Model	Kinetic Parameters	F4	F5	F6	F7	F8	F9
Z-O	k_0_ [mg × min^−1^]	0.71 ± 0.11	0.79 ± 0.12	0.89 ± 0.14	1.50 ± 0.19	1.43 ± 0.22	1.34 ± 0.96
	t_0.5_ [min]	67 ± 14	97 ± 15	114 ± 16	268 ± 35	265 ± 42	295 ± 22
	R^2^	0.59 ± 0.20	0.76 ± 0.02	0.64 ± 0.27	0.75 ± 0.06	0.71 ± 0.05	0.90 ± 0.03
F-O	k_1_ × 10^3^ [min^−1^]	5.59 ± 0.96	4.71 ± 0.33	2.99 ± 0.44	5.40 ± 0.50	5.52 ± 0.55	3.56 ± 0.97
	t_0.5_ [min]	125 ± 22	151 ± 12	254 ± 40	134 ± 10.0	128 ± 12	195 ± 5
	R^2^	0.83 ± 0.04	0.96 ± 0.04	0.86 ± 0.07	0.94 ± 0.07	0.94 ± 0.04	0.99 ± 0.03
S-O	k_2_ × 10^4^ [mg^−1^ × min^−1^]	3.96 ± 0.57	1.31 ± 0.19	0.432 ± 0.070	0.36 ± 0.10	0.372 ± 0.088	0.115 ± 0.013
	t_0.5_ [min]	31 ± 5	61 ± 9	162 ± 28	50 ± 9	42 ± 8	113 ± 14
	R^2^	0.87 ± 0.09	0.88 ± 0.09	0.84 ± 0.09	0.77 ± 0.20	0.76 ± 0.10	0.91 ± 0.02
H	k_H_ [mg × min^−1/2^]	3.44 ± 0.55	6.61 ± 0.51	6.71 ± 0.79	38.9 ± 2.6	37.5 ± 3.4	33.9 ± 1.1
	t_0.5_ [min]	163 ± 55	128 ± 20	209 ± 56	107 ± 14	102 ± 19	135 ± 9
	R^2^	0.67 ± 0.16	0.96 ± 0.01	0.91 ± 0.07	0.95 ± 0.01	0.93 ± 0.02	0.95 ± 0.01
K-P	k_K-P_ × 10^2^ [min^−n^]	6.69 ± 0.84	2.63 ± 0.88	2.97 ± 0.70	1.78 ± 0.18	1.48 ± 0.24	1.42 ± 0.16
	t_0.5_ [min]	39 ± 9	97 ± 50	102 ± 52	131 ± 21	131 ± 30	171 ± 29
	R^2^	0.99 ± 0.01	0.97 ± 0.01	0.96 ± 0.01	0.99 ± 0.01	0.99 ± 0.01	0.99 ± 0.01
	n	0.56 ± 0.04	0.65 ± 0.10	0.64 ± 0.11	0.69 ± 0.03	0.72 ± 0.04	0.70 ± 0.03
H-C	k_H-C_ × 10^3^ [mg^1/3^ × min^−1^]	4.97 ± 0.99	5.68 ± 0.53	4.27 ± 0.68	11.11 ± 0.97	11.20 ± 1.1	8.39 ± 0.20
	t_0.5_ [min]	184 ± 37	195 ± 19	292 ± 49	175 ± 14	169 ± 16	277 ± 6
	R^2^	0.79 ± 0.05	0.94 ± 0.03	0.84 ± 0.07	0.95 ± 0.04	0.94 ± 0.03	0.99 ± 0.03
best fit		K-P	K-P	K-P	K-P	K-P	F-O, K-P; H-C

Z-O—zero-order model; F-O—first order model, S-O—second-order model; H—Higuchi model; K-P—Korsmeyer-Peppas model, H-C—Hixon-Crowell model.

## Data Availability

The data supporting the reported results are available in the Department of Physical Chemistry and Biophysics of Wrocław Medical University.
